# Supplementary material for: To weight or not to weight? Studying the effect of selection bias in three large EHR-linked biobanks
Source: medRxiv. 2024 Feb 13:2024.02.12.24302710. Preprint. [Version 1] doi: 10.1101/2024.02.12.24302710 (PMC10888982; doi:10.1101/2024.02.12.24302710)
Supplement: Supplement 1 [file media-1.pdf]

Supplementary material to…

To weight or not to weight? Studying the effect of selection bias in three EHR-linked biobanks

Maxwell Salvatore, Ritoban Kundu, Xu Shi, Christopher R Friese, Seunggeun Lee, Lars G Fritsche, Alison M Mondul, David Hanauer, Celeste Leigh Pearce, Bhramar Mukherjee

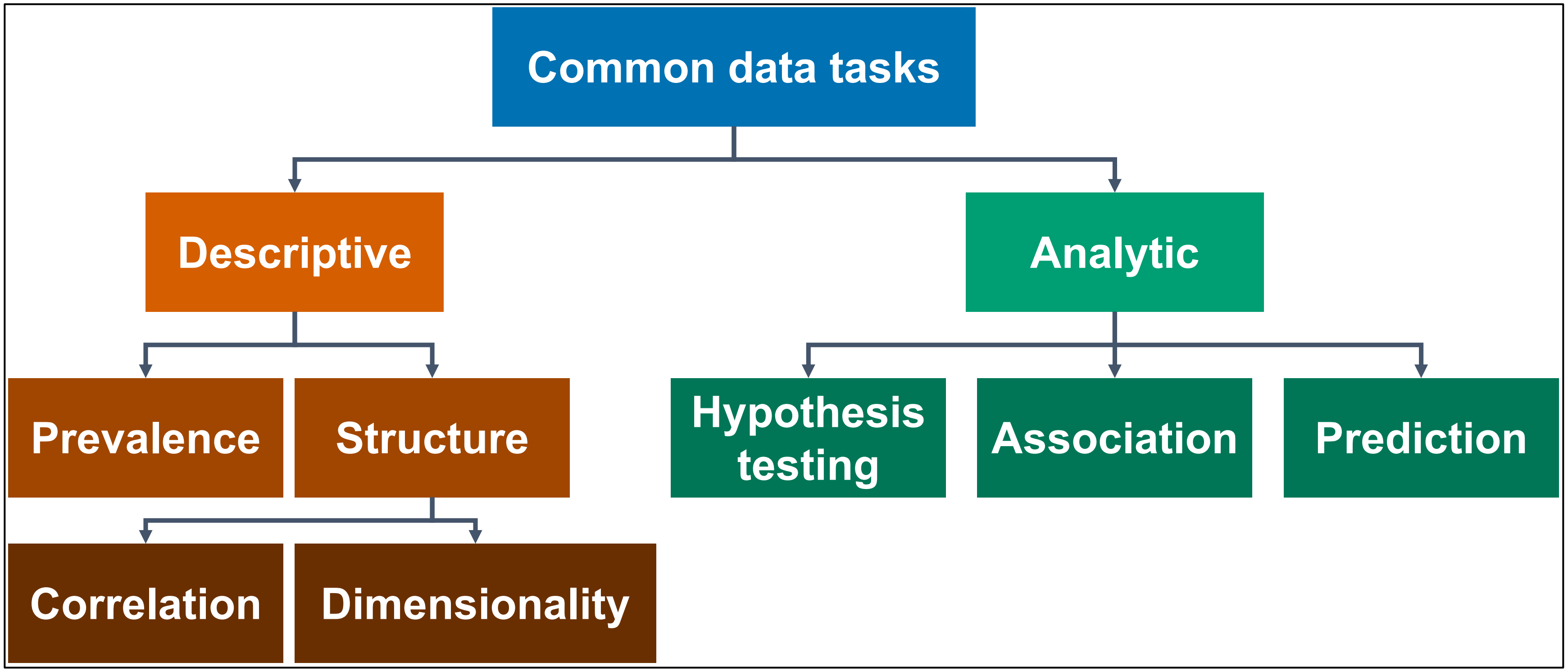

Figure S1. Flowchart depicting several common data tasks. This flowchart is subjective and not exhaustive.

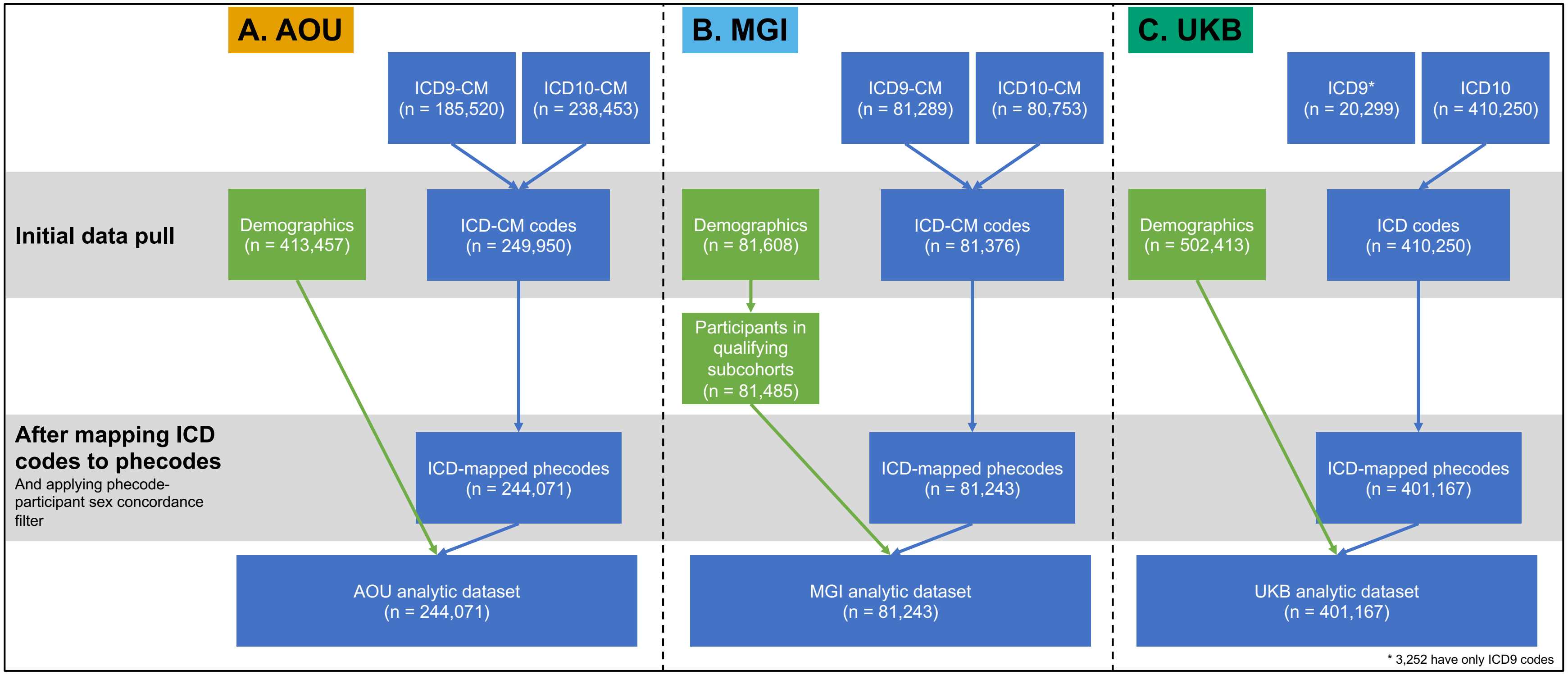

Figure S2. Flowcharts depicting samples sizes before and after filter and ICD-to-phecode mapping in AOU (panel A), MGI (panel B), and UKB (panel C).

Table S1. Phenotypes defined in paper and their qualifying phecode definitions

Variable	Phecode	Description
Anxiety	MB_288	Anxiety and anxiety disorders
Cancer	CA_100	Malignant neoplasm of the head and neck
	CA_100.1	Malignant neoplasm of the oral cavity
	CA_100.12	Malignant neoplasm of the tongue
	CA_100.13	Malignant neoplasm of the gums
	CA_100.14	Malignant neoplasm of the floor of mouth
	CA_100.15	Malignant neoplasm of the palate
	CA_100.2	Malignant neoplasm of the oropharynx
	CA_100.3	Malignant neoplasm of the nasopharynx
	CA_100.4	Malignant neoplasm of the hypopharynx
	CA_100.5	Malignant neoplasm of nasal cavities, middle ear, and accessory sinuses
	CA_100.6	Malignant neoplasm of the larynx
	CA_100.7	Malignant neoplasm of the pharynx
	CA_100.8	Malignant neoplasm of the lip
	CA_100.9	Malignant neoplasm of the salivary glands
	CA_101	Malignant neoplasm of the digestive organs
	CA_101.1	Malignant neoplasm of the esophagus
	CA_101.2	Malignant neoplasm of stomach
	CA_101.21	Malignant neoplasm of cardia
	CA_101.3	Malignant neoplasm of the small intestine
	CA_101.4	Malignant neoplasm of the lower GI tract
	CA_101.41	Colorectal cancer
	CA_101.411	Malignant neoplasm of colon
	CA_101.412	Malignant neoplasm of appendix
	CA_101.42	Malignant neoplasm of anus
	CA_101.6	Malignant neoplasm of the liver and intrahepatic bile ducts
	CA_101.61	Malignant neoplasm of the liver
	CA_101.62	Malignant neoplasm of the intrahepatic bile ducts
	CA_101.7	Malignant neoplasm of the gallbladder and extrahepatic bile ducts
	CA_101.71	Malignant neoplasm of the gallbladder
	CA_101.8	Malignant neoplasm of the pancreas
	CA_102	Malignant neoplasm of the thoracic and respiratory organs
	CA_102.1	Malignant neoplasm of the of bronchus and lung
	CA_102.3	Malignant neoplasm of the trachea
	CA_102.5	Malignant neoplasm of the heart, mediastinum, thymus, and pleura
	CA_102.51	Malignant neoplasm of the heart
	CA_102.52	Malignant neoplasm of the mediastinum
	CA_102.53	Malignant neoplasm of the of pleura
	CA_102.54	Malignant neoplasm of the thymus
	CA_103	Malignant neoplasm of the skin
	CA_103.1	Melanomas of skin
	CA_103.2	Keratinocyte carcinoma
	CA_103.21	Basal cell carcinoma
	CA_103.22	Squamous cell carcinoma of the skin
	CA_103.3	Carcinoma in situ of skin
	CA_104	Malignant sarcoma-related cancers
	CA_104.1	Malignant neoplam of the bone and/or cartilage
	CA_104.2	Malignant neoplasm of retroperitoneum and peritoneum
	CA_104.3	Malignant neoplasm of connective and soft tissue
	CA_104.4	Malignant neoplasm of peripheral nerves*
	CA_104.5	Gastrointestinal stromal tumor*
	CA_104.6	Kaposi's sarcoma

Table S1. Phenotypes defined in paper and their qualifying phecode definitions

Variable	Phecode	Description
	CA_105	Malignant neoplasm of the breast
	CA_105.1	Malignant neoplasm of the breast, female
	CA_105.2	Malignant neoplasm of the breast, male
	CA_106	Gynecological malignant neoplasms
	CA_106.1	Malignant neoplasm of external female genital organs and cervix
	CA_106.11	Malignant neoplasm of the vulva
	CA_106.12	Malignant neoplasm of the vagina
	CA_106.13	Malignant neoplasm of the cervix
	CA_106.2	Malignant neoplasm of the uterus
	CA_106.21	Malignant neoplasm of endometrium
	CA_106.3	Malignant neoplasm of the ovary
	CA_106.4	Malignant neoplasm of the fallopian tube and uterine adnexa
	CA_106.6	Malignant neoplasm of the placenta
	CA_107	Malignant neoplasm of male genitalia
	CA_107.1	Malignant neoplasm of the penis
	CA_107.2	Malignant neoplasm of the prostate
	CA_107.3	Malignant neoplasm of the testis
	CA_107.4	Malignant neoplasm of epididymis
	CA_107.5	Malignant neoplasm of spermatic cord
	CA_107.6	Malignant neoplasm of the scrotum
	CA_108	Malignant neoplasm of the urinary tract
	CA_108.4	Malignant neoplasm of the kidney
	CA_108.41	Malignant neoplasm of kidney, except pelvis
	CA_108.42	Malignant neoplasm of renal pelvis
	CA_108.5	Malignant neoplasm of the bladder
	CA_108.6	Malignant neoplasm of urethra
	CA_108.7	Malignant neoplasm of ureter
	CA_109	Malignant neoplasm of the eye, brain and other parts of central nervous system
	CA_109.1	Malignant neoplasm of eye
	CA_109.11	Malignant neoplasm of orbit
	CA_109.12	Malignant neoplasm of lacrimal gland and duct
	CA_109.13	Malignant neoplasm of conjunctiva
	CA_109.14	Malignant neoplasm of cornea
	CA_109.15	Malignant neoplasm of retina
	CA_109.16	Malignant neoplasm of choroid
	CA_109.2	Malignant neoplasm of meninges
	CA_109.3	Malignant neoplasm of brain
	CA_109.4	Malignant neoplasm of spinal cord
	CA_109.5	Malignant neoplasm of cranial nerve
	CA_110	Malignant neoplasm of the endocrine glands
	CA_110.1	Malignant neoplasm of the thyroid
	CA_110.3	Malignant neoplasm of the parathyroid gland
	CA_110.4	Malignant neoplasm of the pituitary gland and craniopharyngeal duct
	CA_110.5	Malignant neoplasm of the pineal gland
	CA_112	Malignant neoplasm of other and ill-defined sites
	CA_112.1	Mesothelioma*
	CA_114	Neuroendocrine tumors
	CA_114.1	Malignant neuroendocrine tumors
	CA_114.11	Exocrine pancreatic cancer
	CA_114.12	Merkel cell carcinoma
	CA_114.2	Benign neuroendocrine tumors
	CA_114.4	Carcinoid tumors
	CA_114.41	Intestinal carcinoid

Table S1. Phenotypes defined in paper and their qualifying phecode definitions

Variable	Phecode	Description
	CA_114.42	Carcinoid tumor of the bronchus and lung
	CA_114.43	Carcinoid tumor of the thymus
	CA_114.44	Carcinoid tumor of the stomach
	CA_114.45	Carcinoid tumor of the kidney
	CA_114.5	Paraganglioma
	CA_114.6	Pheochromocytoma
	CA_116	Secondary malignant neoplasm
	CA_120	Hemo onc - by cell of origin
	CA_120.1	Myeloid
	CA_120.11	Plasma cell
	CA_120.12	Monocyte
	CA_120.13	Erythroid
	CA_120.14	Megakaryoblast
	CA_120.15	Mast cell
	CA_120.2	Lymphoid
	CA_120.21	Mature B-cell
	CA_120.22	Mature T-Cell
	CA_120.3	Histocytoces
	CA_121	Leukemia
	CA_121.1	Acute leukemia
	CA_121.11	Acute lymphoid leukemia
	CA_121.12	Acute myeloid leukemia
	CA_121.2	Chronic leukemia
	CA_121.21	Chronic lymphoid leukemia
	CA_121.22	Chronic myloid leukemia
	CA_121.23	Chronic myelomonocytic (monocytic) leukemia
	CA_122	Lymphoma
	CA_122.1	Hodgkin lymphoma
	CA_122.11	Nodular sclerosis Hodgkin lymphoma
	CA_122.2	Non-Hodgkin lymphoma
	CA_122.21	Follicular lymphoma
	CA_122.22	Diffuse large B-cell lymphoma*
	CA_122.23	Burkitt lymphoma
	CA_122.24	T-cell lymphoma
	CA_122.25	Anaplastic large cell lymphoma
	CA_122.26	Extranodal NK/T-cell lymphoma, nasal type*
	CA_123	Multiple myeloma and malignant plasma cell neoplasms
	CA_123.1	Multiple myeloma
	CA_124	Myeloproliferative disorder
	CA_124.3	Polycythemia vera
	CA_124.5	Essential thrombocythemia
	CA_124.6	Myelodysplastic syndrome
	CA_124.7	Chronic myeloproliferative disease*
	CA_124.8	Myelofibrosis
	CA_125	Other malignant neoplasms of lymphoid, hematopoietic and related tissue
	CA_128	Estrogen receptor status
	CA_128.1	Estrogen receptor postitive status [ER+]
	CA_128.2	Estrogen receptor negative status [ER-]
	CA_130	Cancer (solid tumor, excluding BCC)
	CA_132	Sequelae of cancer
Coronary artery disease	CV_404.2	Coronary atherosclerosis [Atherosclerotic heart disease]
Depression	MB_286.2	Major depressive disorder

Table S1. Phenotypes defined in paper and their qualifying phecode definitions

Variable	Phecode	Description
Diabetes	EM_202	Diabetes mellitus

Visit https://phewascatalog.org (phecodeX) and https://github.com/PheWAS/PhecodeX

Table S2. Definition of variables by cohort used throughout paper

	AOU	MGI	UKB	NHIS (2019)*
Age	Age at last diagnosis	Age at last diagnosis	Age at consent: date of consent (field ID 200) minus date of birth (field IDs 34, 52)	Age at screening (AGEP_A)
Sex	Self-reported sex at birth (field name: sex_at_birth_concept_id)	Self-report EHR	Acquired by central registry at recruitment, may be updated by individual (field ID 31)	SEX_A
Race/ethnicity	Self-reported race ethnicity (field names: race_source_concept_id, ethnicity_source_concept_id)	Self-report EHR	Self-report survey (field ID 21000)	HISPALLP_A
BMI	Median of EHR values	Median of EHR values	Median of assessed values (field ID 21001)	BMICAT_A (HEIGHTTC_A, WEIGHTLBTC_A)
Smoking status	Self-report (concept IDs: 1585857, 1585860)	Self-report EHR	Survey (field ID 20116)	SMKCIGST_A
Anxiety	Phecode MB_288: Anxiety and anxiety disorders			GADCAT_A
Cancer	See Table S1			CANEV_A
Coronary artery disease	Phecode CV_404.2: Coronary atherosclerosis [Atherosclerotic heart disease]			CHDEV_A
Depression	Phecode MB_286.2: Major depressive disorder			PHQCAT_A
Diabetes	Phecode EM_202: Diabetes mellitus			DIBEV_A

* visit https://www.cdc.gov/nchs/nhis/2019nhis.htm for more information

Supplementary Methods

Inverse probability weighting

In MGI, we estimated the first term, $P(S_{external} = 1|\boldsymbol{X})$, by fitting a simplex regression model for the known design probabilities using NHIS data. We estimated the numerator of the second term, $P(S = 1|\boldsymbol{X}, S_{all} = 1)$, using a logistic regression model. We considered the set of selection factors, $\boldsymbol{X}$: age (≥ 50 indicator), female sex, BMI (categorical), non-Hispanic White race/ethnicity, and EHR-derived binary indicators for anxiety, depression, diabetes, cancer, and hypertension (variable definitions in **Table S2**). Cancer was not included directly in the estimation procedure above because the small prevalence of cancer in NHIS led to unstable model fitting.[1] Instead, a cancer factor, γ_{cancer}, defined as $\frac{P(\text{Cancer}|\boldsymbol{X}, S=1)}{P(\text{Cancer}|\boldsymbol{X})}$, was estimated by fitting logistic regression models with the same $\boldsymbol{X}$. The probabilities, ψ, were multiplied by this factor (i.e., $\psi\gamma_{cancer}$).

In AOU, we flexibly selected $\boldsymbol{X}$ by splitting the data in half and fitting a lasso-penalized logistic regression model on $\boldsymbol{X}$ and all possible pairwise interactions using the glmnet R package (version 4.1-8). We considered a set of selection factors, $\boldsymbol{X}$: age ((≥ 50 indicator), female sex, non-Hispanic White race/ethnicity, non-heterosexual orientation (yes/no), health insurance coverage status (yes/no), annual household/family income (≥ $75,000), educational attainment (at least high school graduate or equivalent), and region of residence (indicators for West, South, and Northeast) (variable definitions in **Table S2**). Using 10-fold cross-validation, we selected the largest λ such that the error is within 1 standard error of the minimum to result in a parsimonious model. Of the 55 possible main effect and interaction terms, 39 were selected by this model (**Table S3**) and (along with the main effect for West region) were then used as the final set of $\boldsymbol{X}$ to estimate IP weights in the other half of the data as described for MGI above. The indicator variables for income, health insurance status, and non-Hispanic White race/ethnicity were the three most important variables (**Figure S2**). In both cohorts, the resulting probabilities were winsorized at the 2.5th and 97.5th percentiles.

We note that augmented inverse probability weighting (AIPW) is a doubly robust weighting method that may be of interest to the reader; see [2–4].

Poststratification weighting

In AOU, we considered the set of $\boldsymbol{X}$: age (≥ 50 indicator), female sex, non-Hispanic White race/ethnicity, sexual orientation (non-heterosexual indicator), health insurance coverage status (yes/no), annual household/family income (≥ $75,000 indicator), and region of residence (categorical). In MGI we considered the set of selection factors, $\boldsymbol{X}$: age (≥ 50 indicator), female sex, non-Hispanic White race/ethnicity, BMI (categorical), smoking status (ever/never), and EHR-derived history of anxiety, cancer, depression, diabetes, and hypertension.

We note that other there are other weighting methods relying only on summary statistics like calibration, raking, and pseudo likelihood that may be of interest to the reader; see [5–7].

Correlations
We also explored the correlation structure of unweighted and weighted phenomes through partial correlations. Unweighted partial correlations were calculated between pairs of traits, X and Y, adjusted for age and sex, using the ppcor R package (version 1.1). [8] Weighted partial correlations were approximated as the coefficient β_X from the weighted multiple linear regression model $Y = \beta_0 + \beta_X X + \boldsymbol{\beta_Z Z}$, where X, Y, and $\boldsymbol{Z}$ were mean standardized and $\boldsymbol{Z}$ were age and female sex. For X, Y pairs where one trait was sex-specific, the other trait was limited to individuals of that sex, and sex was not included as a covariate. Network graphs of correlations with absolute values greater than 0.3 were constructed to visually inspect the structure. All traits were treated as binary based on the presence of a single phecode in the EHR. (See **Section S1** for results).

PheWAS
The data were prepared as described in Salvatore and colleagues [9] at the one-year prior to colorectal cancer diagnosis threshold. For sex-specific phecodes, those with discordant sex were treated as missing. (Of note, some ICD codes do not map to phecodes). Logistic regression models were fit as follows:

$$logit\left(P(\mathrm{CA}_{101.41} = 1 | k, \boldsymbol{covariates})\right) = \beta_0 + \beta_k k + \boldsymbol{\beta}_{\mathbf{covariates}}\mathbf{covariates} \qquad \textit{Eq. (S1)}$$

where CA_101.41 (the phecode for colorectal cancer) is an indicator for the outcome, k represents the exposure phecode k (indicator), and covariates are age at one-year prior to colorectal cancer diagnosis (continuous), female sex (indicator), and length of EHR follow-up (continuous).

Phenomewide significant hits were identified using a conservative multiple testing corrected threshold of 0.05 divided by the number of *total* tests. Weighted logistic regression models were fit using svyglm from the survey R package.[10] In cases where a given exposure phecode did not have both (1) at least 20 occurrences and (2) at least 10 individuals with the exposure and colorectal cancer, weighted Firth bias-corrected logistic regression (logistf R package version 1.26.0) was used to address concerns about separation.

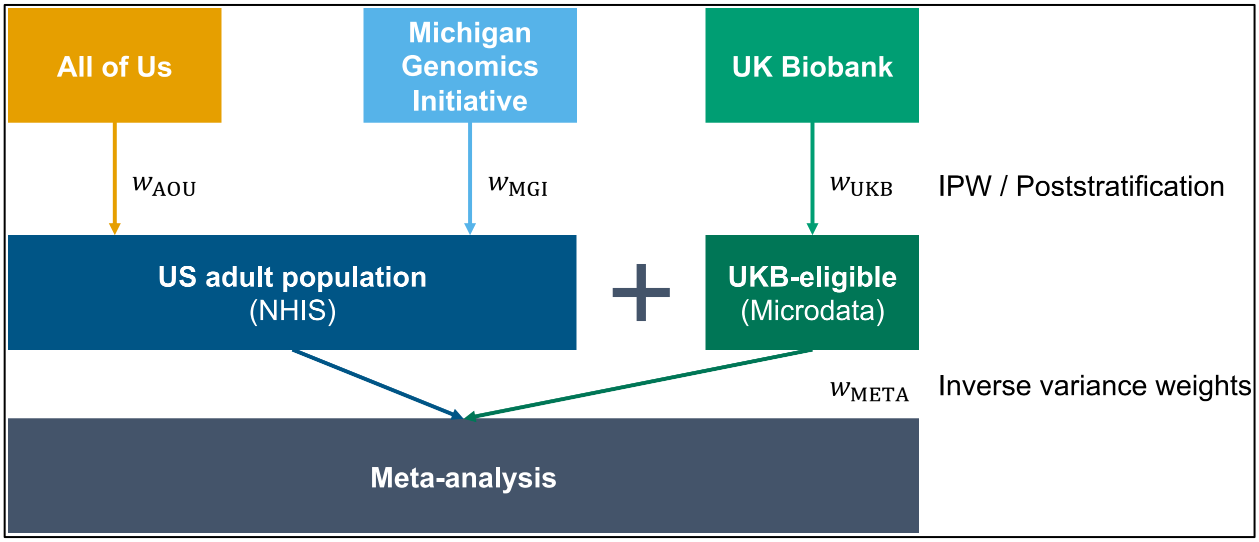

Figure S3. A schematic representation of the targeted and untargeted association analyses pipelines carried out in the manuscript.

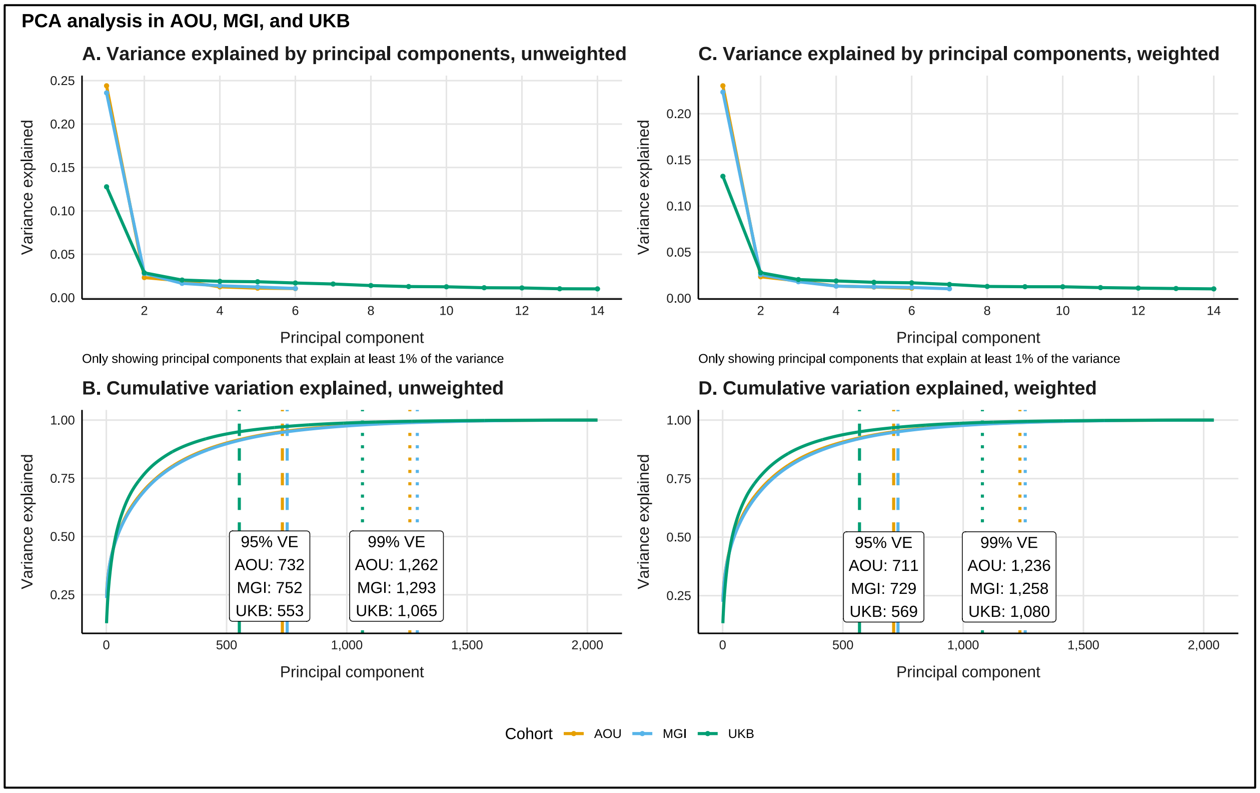

Figure S4. Principal components (PC) analysis in All of Us (AOU), the Michigan Genomics Initiative (MGI), and the UK Biobank (UKB). Panel A shows all principal components explain at least 1% of variation. Panel B shows the cumulative proportion of variance explained (VE) and reports variance explanation thresholds. The vertical dashed lines represent the number of PCs that explain at least 95% of total variance. The vertical dotted lines represent the number of PCs that explain at least 99% of the total variance.

Section S1. Unweighted and weighted partial correlations

Network diagrams depicting unweighted and weighted partial correlation coefficients with absolute values greater than 0.3 (an arbitrary threshold) in AOU is shown in **Figure S5** (MGI and UKB shown in **Figures S6** and **S7**). We can see clusters of correlated traits within endocrine/metabolic and musculoskeletal categories, as well as a cluster including both digestive and neurological traits. A small reduction in correlations with absolute values greater than 0.3 were observed after weighting (2,533 vs. 2,474). Interestingly, we see strong correlations with neoplasm traits in MGI (**Figure S6**), which largely disappear after weighting. There are distinct clusters within musculoskeletal traits and across circulatory system and endocrine/metabolic traits in UKB, which remain after weighting. The number of strong (absolute value > 0.3) correlations in UKB slightly increases after weighting (1,674 vs 1,757). **Figures S8** and **S9** depict the distribution of the unweighted and weighted partial correlation coefficients in each cohort, respectively. Generally, correlations tend to be highest in MGI followed by AOU and then UKB. Comparing the two US-based cohorts, AOU (**Figure S5**) and MGI (**Figure S6**), we see that, while the prevalences of traits involved in these networks are comparable, the network in MGI is denser compared to AOU.

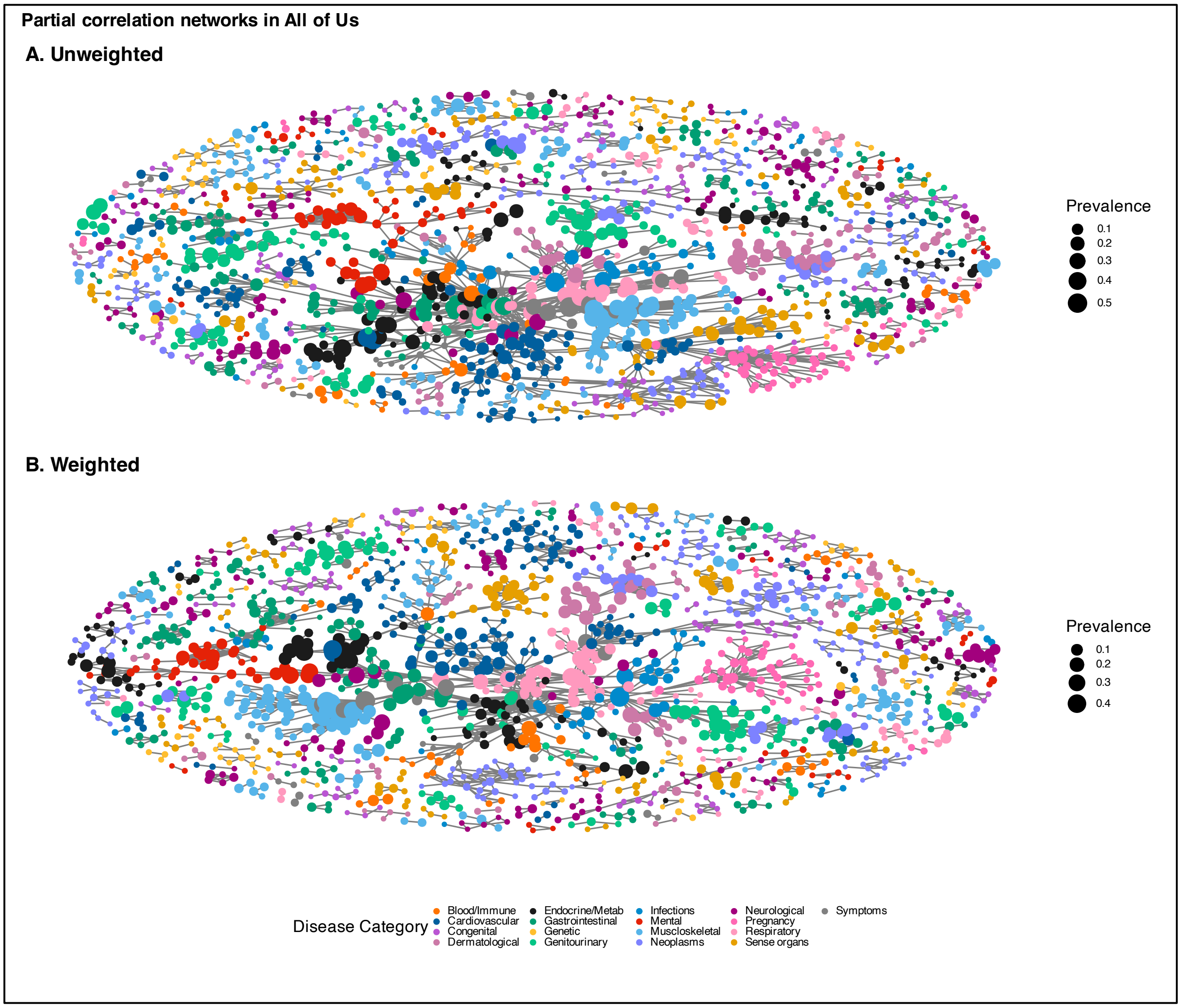

Figure S5. Unweighted (panel A) and inverse probability-weighted (panel B) network plots of the partial correlation structure of medical phenomes in All of Us. Correlation coefficients are adjusted for age and sex. Only correlations with an absolute value greater than or equal to 0.3 are shown. The size of the nodes corresponds to the prevalence of the trait in its cohort and the color corresponds to the phecode category. Corresponding figures for MGI and UKB are in **Figures S6** and **S7**, respectively.

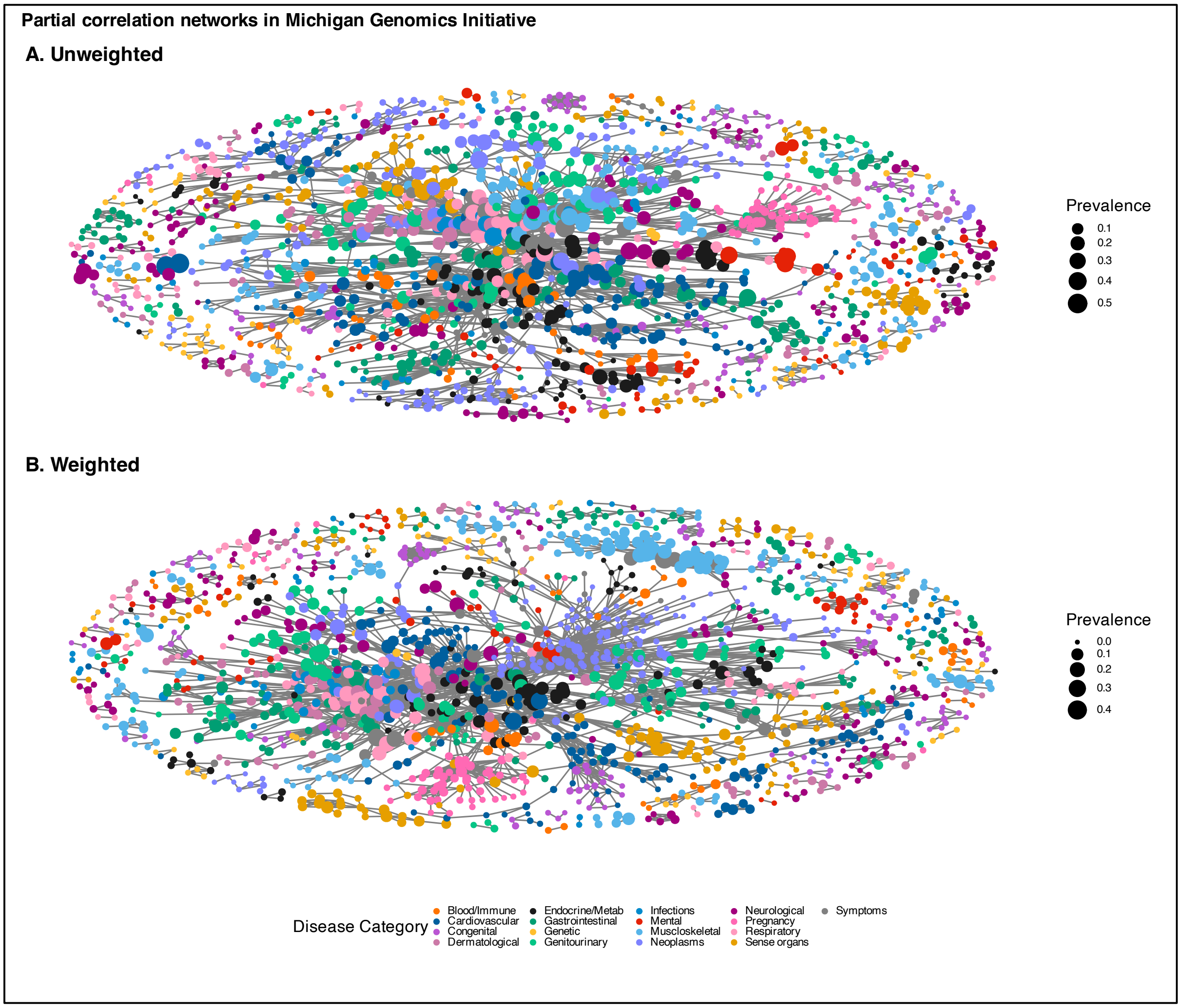

Figure S6. Unweighted (panel A) and inverse probability-weighted (panel B) network plots of the partial correlation structure of medical phenomes in MGI. Correlation coefficients are adjusted for age and sex. Only correlations with an absolute value greater than or equal to 0.3 are shown. The size of the nodes corresponds to the prevalence of the trait in its cohort and the color corresponds to the phecode category. Corresponding figures for AOU and UKB are in **Figures S5** and **S7**, respectively.

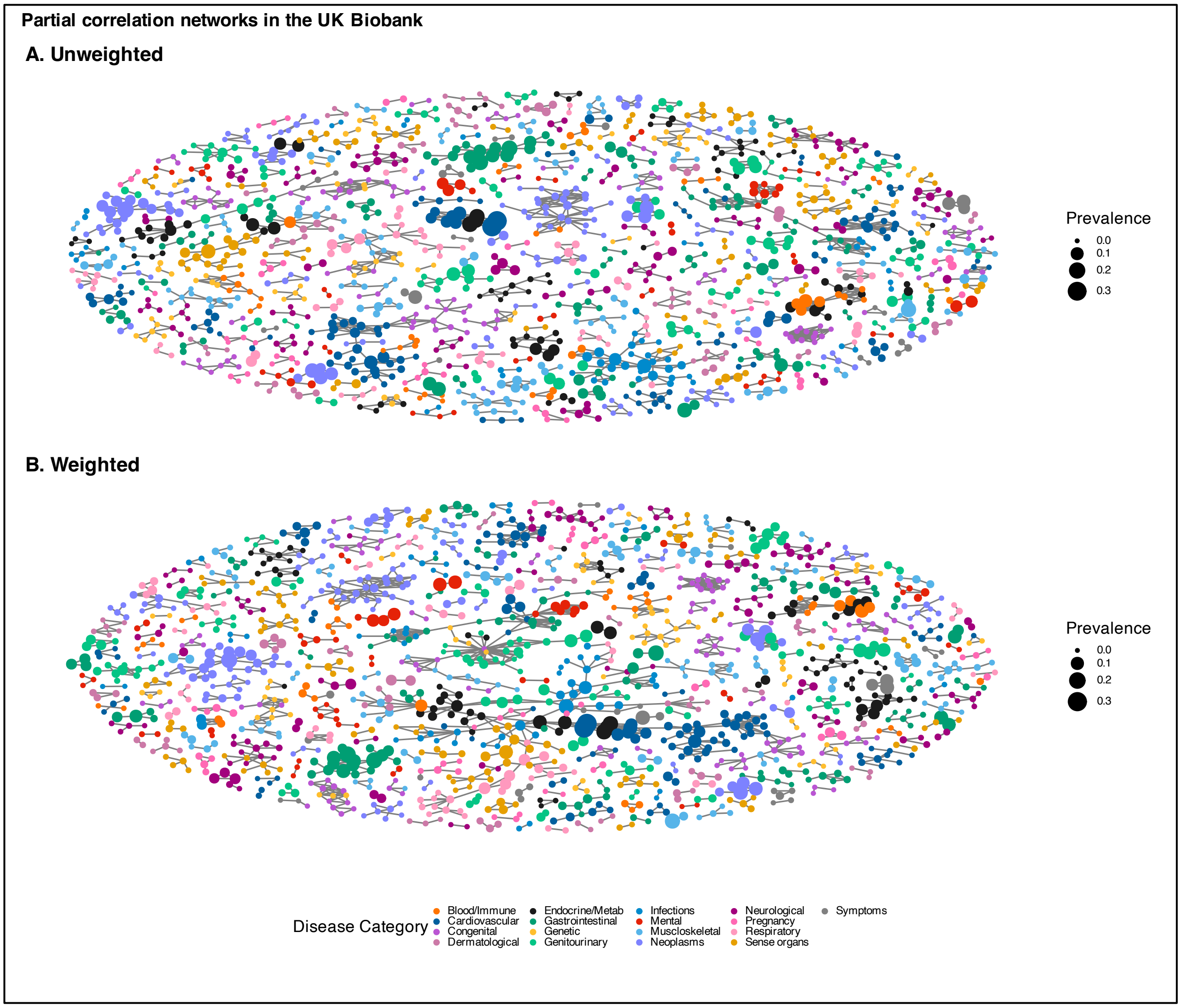

Figure S7. Unweighted (panel A) and inverse probability-weighted (panel B) network plots of the partial correlation structure of medical phenomes in UKB. Correlation coefficients are adjusted for age and sex. Only correlations with an absolute value greater than or equal to 0.3 are shown. The size of the nodes corresponds to the prevalence of the trait in its cohort and the color corresponds to the phecode category. Corresponding figures for AOU and MGI are in **Figures S5** and **S6**, respectively.

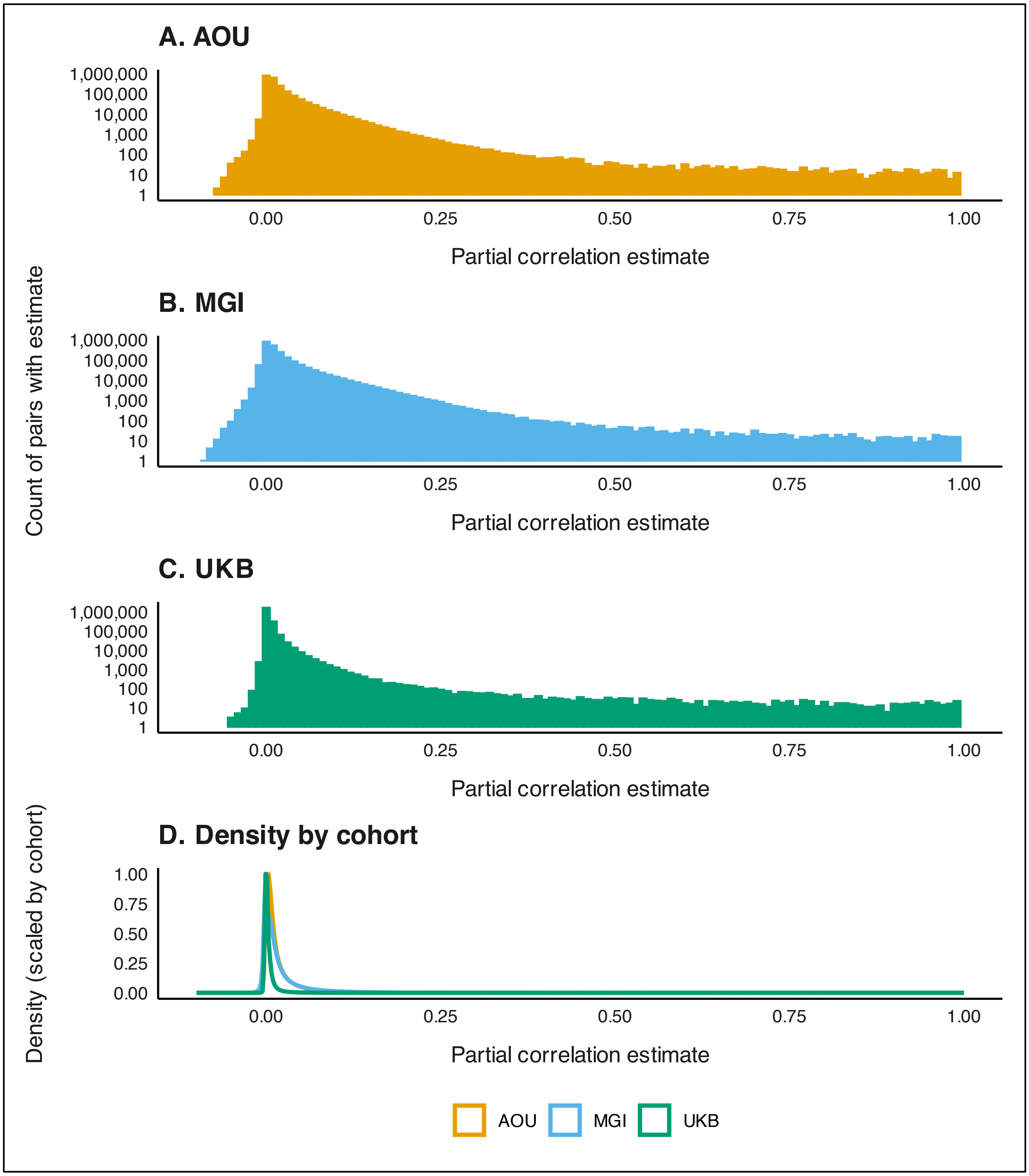

Figure S8. Distribution of unweighted partial correlations across medical phenomes. Partial correlations were adjusted for age and, if both codes in the pair applied to both sexes, sex.

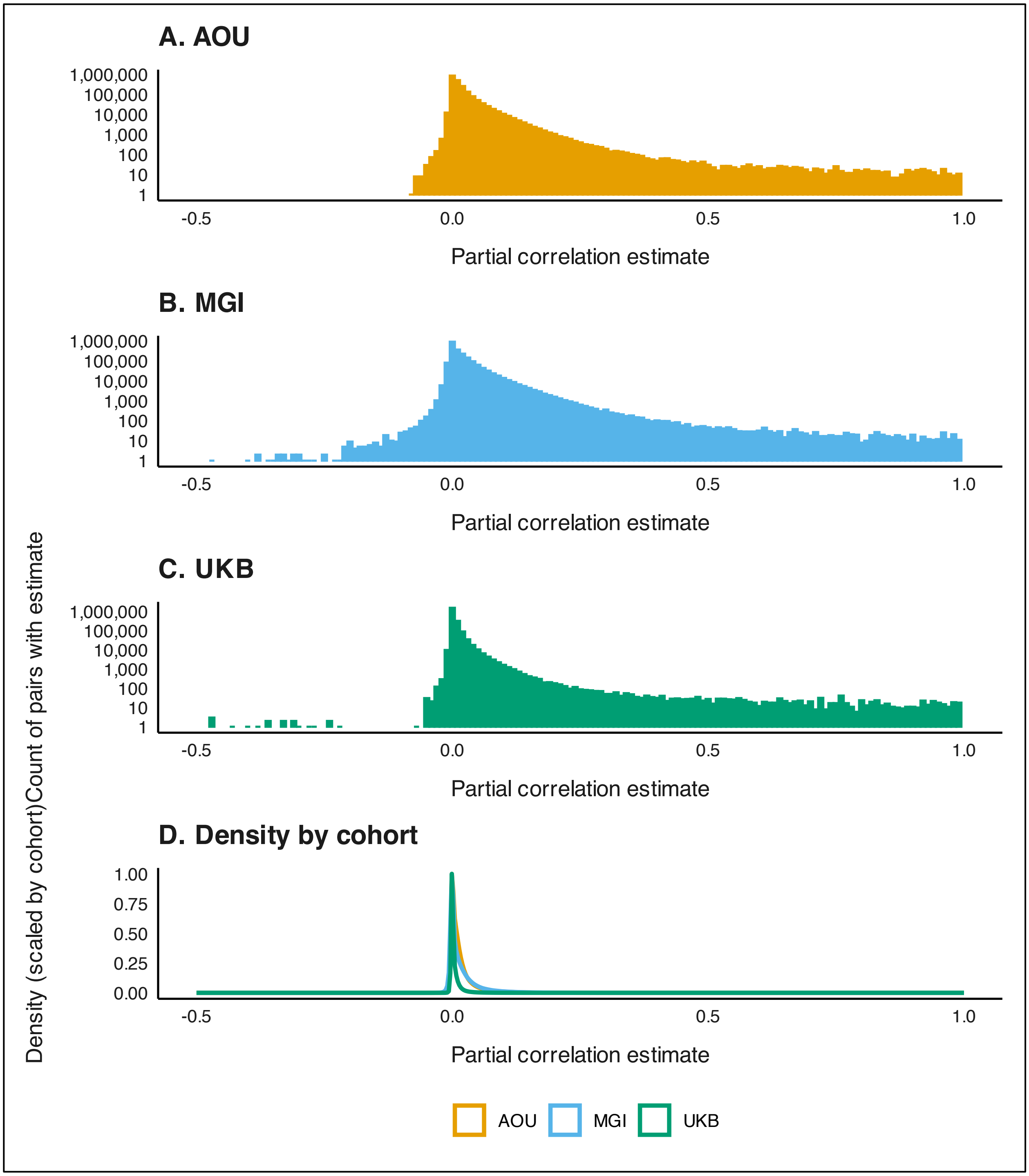

Figure S9. Distribution of weighted partial correlations across medical phenomes. Partial correlations were adjusted for age and, if both codes in the pair applied to both sexes, sex. IP-based weights were used for AOU and MGI and IP-based weighted developed by van Alten and colleagues[11] were used for UKB.

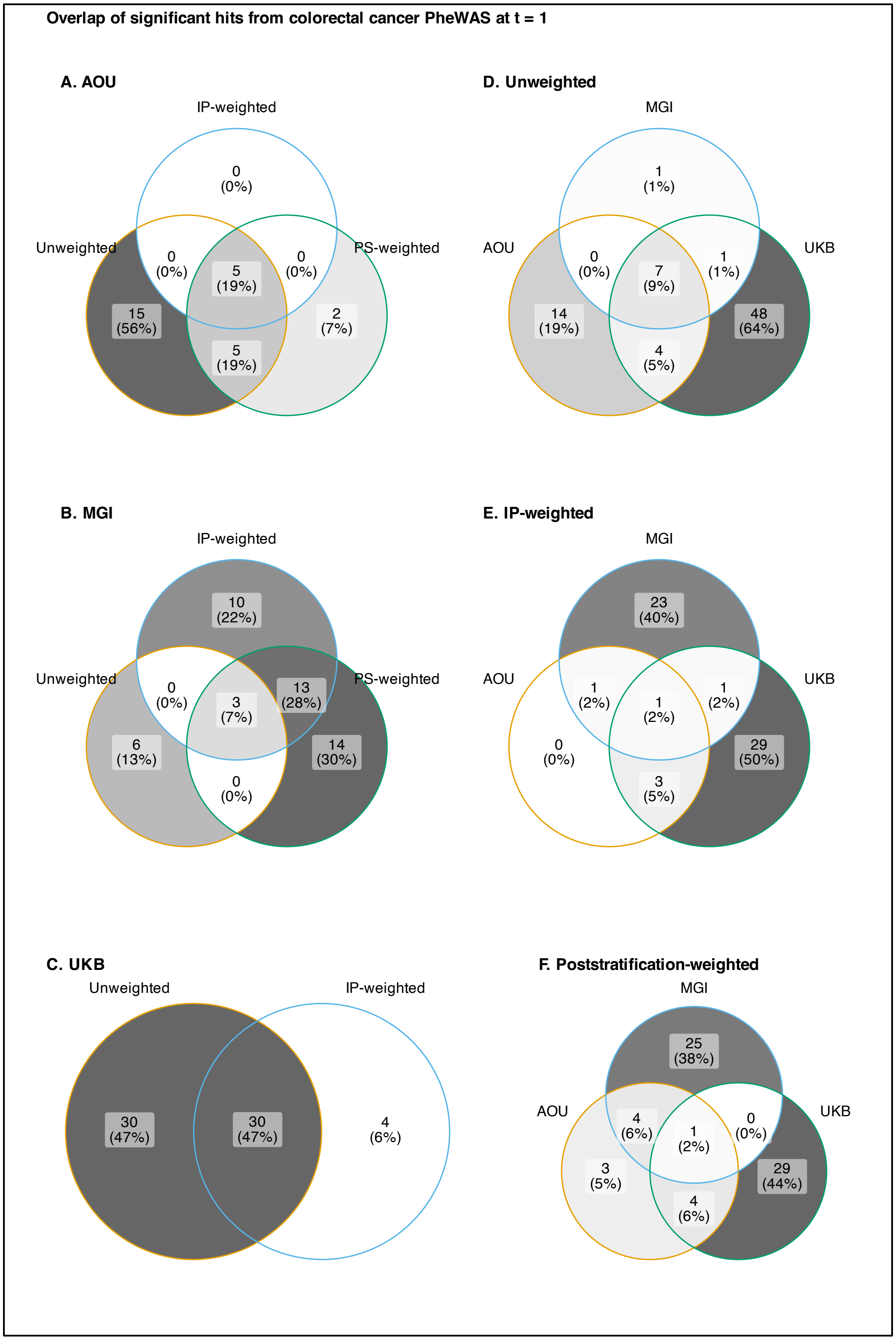

Figure S10. Venn diagrams comparing the overlap in phenome-wide significant hits from unweighted and weighted colorectal cancer PheWAS in AOU, MGI, and UKB.

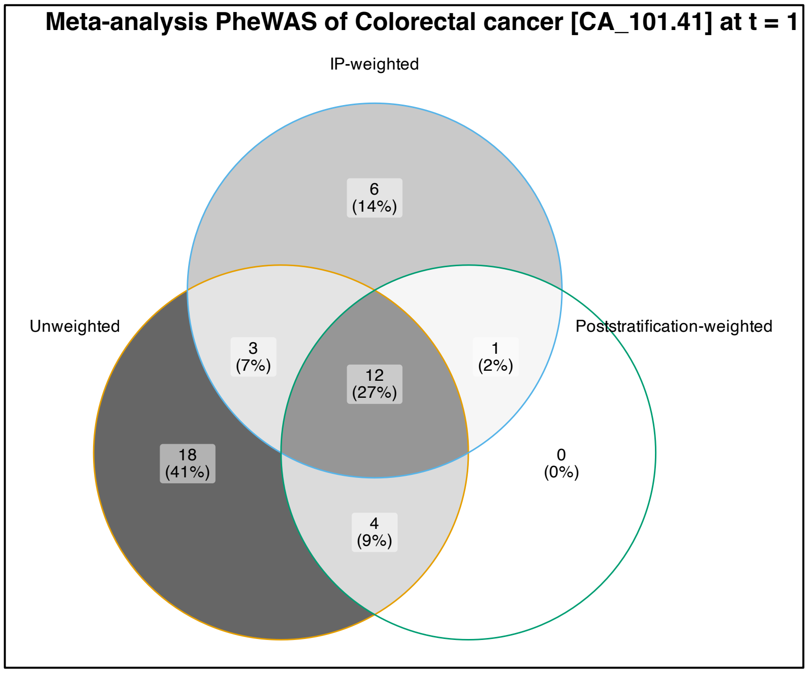

Figure S11. Venn diagrams comparing the overlap in phenome-wide significant hits from meta-analysis PheWAS.

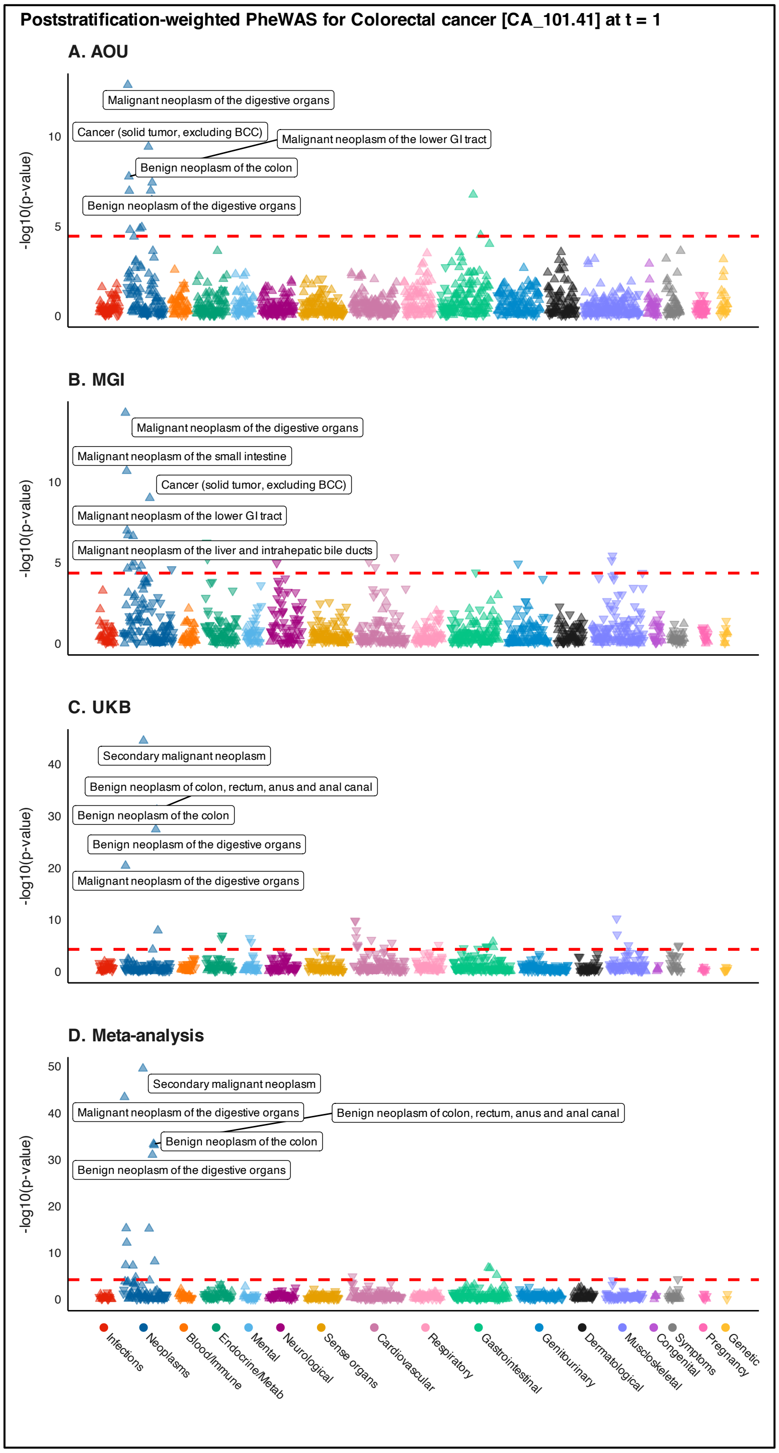

Figure S12. Manhattan plots summarizing poststratification-weight (panels A-D) phenomewide association studies for colorectal cancer in All of Us and the Michigan Genomics Initiative and the inverse probability weighted UK Biobank using 1:2 case:non-case matched data restricted to one year prior to initial diagnosis along with the corresponding meta-analsysis. The dashed red line represents the Bonferroni-corrected p-value threshold (-log10(0.05/number of traits)). The five traits with the smallest p-values are labeled. The upward (downward) orientation of the triangle indicates a positive (negative) association. Plots corresponding to unweighted and IP-weighted PheWAS are presented in **Figure 4**.

Table S3. Female log odds ratio estimate (95% confidence interval) for colorectal cancer (phecode CA_101.41).

Weighting	Covariates	AOU	MGI	UKB	META
Unweighted	None	**-0.287** (-0.354, -0.220)	**-0.303** (-0.387, -0.219)	**-0.450** (-0.492, -0.409)	**-0.390** (-0.423, -0.358)
	Age	**-0.098** (-0.164, -0.033)	**-0.164** (-0.247, -0.082)	**-0.389** (-0.431, -0.348)	**-0.284** (-0.316, -0.252)
IP-weighted	None	-0.037 (-0.188, 0.113)	**-0.266** (-0.467, -0.065)	**-0.443** (-0.506, -0.380)	**-0.373** (-0.429, -0.317)
	Age	-0.047 (-0.198, 0.104)	**-0.217** (-0.419, -0.014)	**-0.398** (-0.461, -0.334)	**-0.335** (-0.392, -0.279)
PS-weighted	None	-0.135 (-0.321, 0.052)	**-0.329** (-0.615, -0.044)	**-0.443** (-0.506, -0.380)	**-0.408** (-0.466, -0.349)
	Age	-0.123 (-0.311, 0.064)	**-0.342** (-0.629, -0.056)	**-0.398** (-0.461, -0.334)	**-0.368** (-0.427, -0.310)

* Meta-analysis results include IP-weighted estimate from UKB
Abbrevs: AOU, All of Us; IP, inverse probability; META, meta-analysis; MGI, Michigan Genomics Initiative; PS, poststratification; UKB, UK Biobank
Bolded point estimates are statistically significant at the 95% confidence level

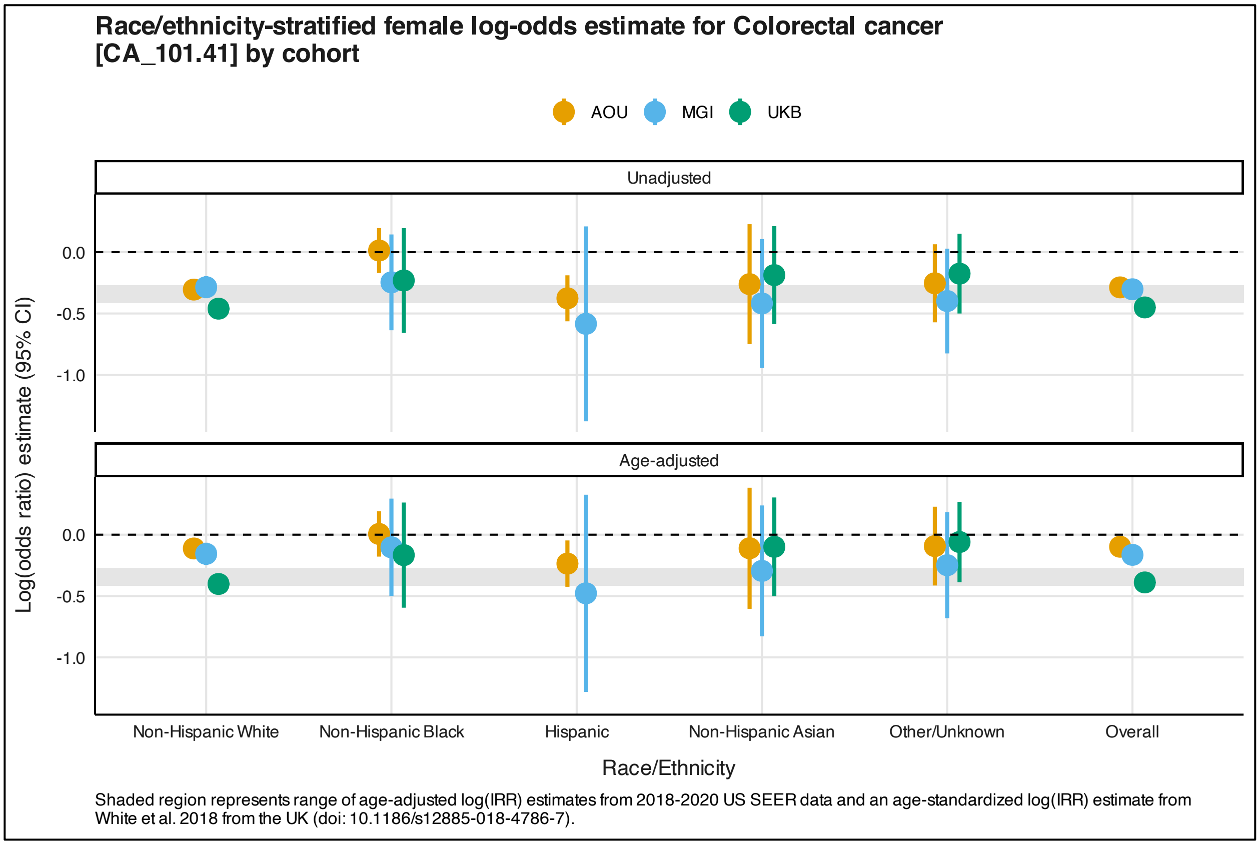

Figure S13. Unadjusted and age-adjusted female log-odds ratio estimate (95% confidence interval) for colorectal cancer (phecode CA_101.41) by race/ethnicity and cohort. Point estimate shapes correspond to the weighting method (circle, unweighted; square, IP-weighted; triangle, PS-weighted). Line colors correspond to the cohort (orange, AOU; blue, MGI; green, UKB). Shaded region represents range of age-adjusted log(incidence rate ratio [IRR]) estimates from 2018-2020 US SEER data and an age-standardized log(IRR) estimate from White et al. 2018 from the UK.[12]

Section S2. Comments on methodological considerations in EHR-based data analysis

Weighting-based analytic approaches present a relatively simple way for researchers to improve the generalizability of their results and help *reduce* (not *remove*) selection bias. IP weights are preferred to PS weights though they rely on the assumption that the weighting model is correctly specified. Regression-based weights can be made more flexible through the use of indicator variables (as in our AOU IP-weights and in van Alten and colleagues[13]), though non-parametric methods like random forest can be used. When individual-level data from the target population is not available, PS weights can be estimated using summary-level strata probabilities (provided these probabilities are conditionally independent). When selection weights are unavailable, methods like covariate or propensity score adjustment, which are simple to implement, can be considered to address in some situations where selection bias is a concern.

Beyond introductory papers,[14–19] substantial work has focused specifically on traditional methodological concerns including confounding,[20,21] misclassification,[7,22,23] missing data,[24–30] and selection bias and cohort representativeness[1,6,7,31–34] related to EHR-based cohorts. For example, traits defined using the phecode framework have demonstrated reduced misclassification compared to ICD codes.[35] One method to further reduce the impact of misclassification, described by Hubbard and colleagues, relies on EHR-derived probabilistic phenotyping.[22] Others have described methods using manual chart review on a subset of data to improve EHR-derived phenotypes.[23,36,37] Beesley and Mukherjee developed three novel likelihood-based bias correction strategies to address outcome misclassification of EHR-derived disease status.[7] Teixeira and colleagues explored incorporation of unstructured data like doctors notes, which improved the identification of hypertensive individuals compared to using ICD codes and blood pressure reading cutoffs alone.[38] Missing data is another issue that has received attention to avoid loss of power and inducing selection bias (via complete case analyses) and aid in meeting assumptions necessary for multiple imputation.[25] One avenue is using non-missing genotype data available in EHR-linked biobanks to inform imputation, which demonstrated improvements in imputation of cardiovascular related measurements.[39] This idea could be extended using exposure polygenic risk scores[40] to inform imputation of missing exposure data.

One consideration broadly applicable in health research but is particularly acute in EHR-based analyses is *target validity*. Westreich and colleagues have defined this as a joint measure of internal and external validity of an effect estimate with respect to a specific target population.[41] Historically, internal validity, the notion that an estimate reflects the true underlying parameter in the study population, has taken precedence over external validity, that the parameter in the study population is representative of the true parameter in the target population. However, because of observation mechanisms and recruitment strategies into EHR-linked biobanks, the target population is almost certainly never (1) exactly the study sample or (2) the population of which the study sample is a simple random sample.[41] EHR researchers should think critically regarding

who the results are intended for or representative of before beginning an analysis and make their target populations explicit in their work. We believe it is critical for researchers to consider weighted approaches that account for both the observation and recruitment mechanisms in each cohort (including potential subcohorts) and differences in the distribution of key characteristics between the analytic cohort and the target population.

We want to highlight some considerations that are hallmarks of EHR analysis. One such consideration is *informed presence*, defined by Goldstein and colleagues as "the notion that inclusion in an EHR is not random but rather indicates that the subject is ill, making people in EHRs systematically different from those not in EHRs."[42] This resulting discrepancy harms generalizability to general populations who tend to be healthier than those in the EHR data sample and results in bias. This concept extends to individuals within the EHR – those that are sicker tend to have more encounters and records than those who are healthier – and, in some cases, to records in the EHR (e.g., lab results). This phenomenon is illustrated by Agniel and colleagues, which shows that the presence and timing of laboratory results was more informative than the value of the laboratory results themselves. [43] Interested readers can learn more about informed presence elsewhere. [14,42,44–46] Including EHR metadata, like length of follow-up, number of encounters, density of laboratory measurements, and visit type (e.g., outpatient vs inpatient vs emergency), and careful selection or matching of controls in analyses are recommended to improve exchangeability and attempt to make EHR observation mechanisms comparable.

Section S3. Investigation into infectious diseases peak in AOU PheWAS using phecode 1.2 mapping tables

An earlier version of the manuscript was performed using the phecode 1.2 mapping tables instead of phecode X. The Manhattan plot representing the colorectal cancer PheWAS in AOU in **Figure S14A** shosw a peak in the infectious disease category. The top hit is Human immunodeficiency virus [HIV] disease, or phecode 071 in the phecode 1.2 mapping tables. It is well established that there is no association between HIV status and colorectal cancer.[47,48] We investigated the underlying ICD codes that are qualify as a colorectal cancer case. Our analyses in the manuscript use the phecode mapping table present in the PheWAS R package (version 1.2).[49,50] We also show qualifying ICD codes for a different phecode mapping table (version X),[51,52] which defines over 3,600 traits. The results of the differences in qualifying ICD codes, number of individuals with the ICD code, and the number (and percent) overlap with individuals who have HIV according to their version 1.2 defined phecode are summarized in **Table S5**. We see that there is significant overlap between individuals with ICD codes for anal Pap smears, inconclusive results and *carcinoma in situ* and HIV status. These codes are present in the version 1.2 mapping table, but not in the version X mapping table. Codes present in the version 1.2 definition also include malignant neoplasms of the anus, but not in the version X definition. And there is evidence that people living with HIV experience higher incidence of anal cancer.[53] Because version X has more traits, there is greater separation between colorectal cancer and anal cancer.

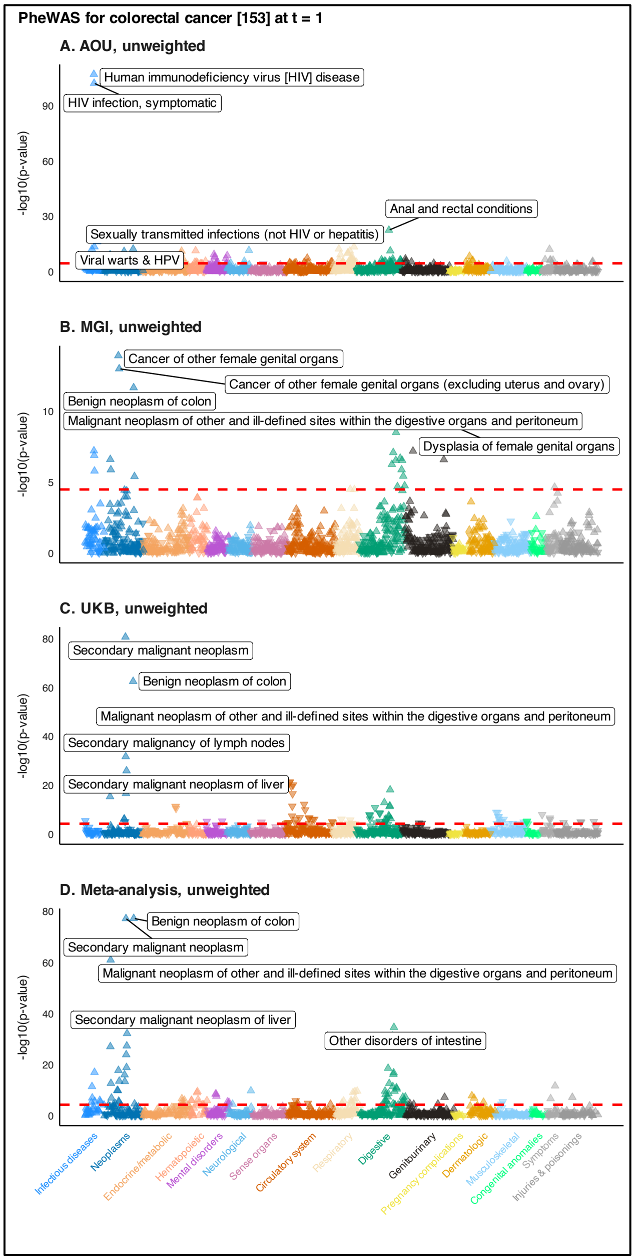

Figure S14. Manhattan plots summarizing unweighted (panels A-C) phenomewide association studies for colorectal cancer in All of Us, the Michigan Genomics Initiative, and UK Biobank using 1:2 case:non-case matched data restricted to one year prior to initial diagnosis. Panel D shows the unweighted meta-analysis PheWAS, respectively. The dashed red line represents the Bonferroni-corrected p-value threshold (-log10(0.05/number of traits)). The five traits with the smallest p-values are labeled. The upward (downward) orientation of the triangle indicates a positive (negative) association.

Table S5. Comparison between ICD codes by colorectal cancer phecode mapping table, count with ICD code, and overlap with individuals who have HIV phecode (sorted by proportion of overlap).

ICD Code	ICD Version	ICD Code Description	Qualifying ICD codes for colorectal cancer phecode by mapping table version		Number with qualifying ICD code	Number with ICD codes and HIV phecode	Percent
			Version 1.2	Version X			
796.71	ICD9CM	Papanicolaou smear of anus with atypical squamous cells of undetermined significance (ASC-US)	1	0	87	74	85.1
796.74	ICD9CM	Papanicolaou smear of anus with high grade squamous intraepithelial lesion (HGSIL)	1	0	29	23	79.3
796.73	ICD9CM	Papanicolaou smear of anus with low grade squamous intraepithelial lesion (LGSIL)	1	0	100	79	79
230.6	ICD9CM	Carcinoma in situ of anus, unspecified	1	0	70	53	75.7
R85.613	ICD10CM	High grade squamous intraepithelial lesion on cytologic smear of anus (HGSIL)	1	0	61	45	73.8
R85.612	ICD10CM	Low grade squamous intraepithelial lesion on cytologic smear of anus (LGSIL)	1	0	166	118	71.1
796.7	ICD9CM	Abnormal glandular Papanicolaou smear of anus	1	0	124	86	69.4
R85.610	ICD10CM	Atypical squamous cells of undetermined significance on cytologic smear of anus (ASC-US)	1	0	155	105	67.7
230.5	ICD9CM	Carcinoma in situ of anal canal	1	0	74	50	67.6
R85.619	ICD10CM	Unspecified abnormal cytological findings in specimens from anus	1	0	103	61	59.2
D01.3	ICD10CM	Carcinoma in situ of anus and anal canal	1	0	219	129	58.9
796.72	ICD9CM	Papanicolaou smear of anus with atypical squamous cells cannot exclude high grade squamous intraepithelial lesion (ASC-H)	1	0	15	8	53.3
C7A.024	ICD10CM	Malignant carcinoid tumor of the descending colon	1	0	2	1	50
R85.611	ICD10CM	Atypical squamous cells cannot exclude high grade squamous intraepithelial lesion on cytologic smear of anus (ASC-H)	1	0	29	14	48.3
796.76	ICD9CM	Papanicolaou smear of anus with cytologic evidence of malignancy	1	0	3	1	33.3
154.3	ICD9CM	Malignant neoplasm of anus, unspecified site	1	0	107	20	18.7
C21.0	ICD10CM	Malignant neoplasm of anus, unspecified	1	0	163	28	17.2
209.1	ICD9CM	Malignant carcinoid tumor of the large intestine, unspecified portion	1	0	7	1	14.3
C21.1	ICD10CM	Malignant neoplasm of anal canal	1	0	96	13	13.5
D37.5	ICD10CM	Neoplasm of uncertain behavior of rectum	0	1	16	2	12.5
154.2	ICD9CM	Malignant neoplasm of anal canal	1	0	56	6	10.7
154.8	ICD9CM	Malignant neoplasm of other sites of rectum, rectosigmoid junction, and anus	1	0	30	2	6.7
C21.8	ICD10CM	Malignant neoplasm of overlapping sites of rectum, anus and anal canal	1	0	45	3	6.7
153.4	ICD9CM	Malignant neoplasm of cecum	1	1	71	4	5.6
154.1	ICD9CM	Malignant neoplasm of rectum	1	1	204	11	5.4
C18.8	ICD10CM	Malignant neoplasm of overlapping sites of colon	1	1	45	2	4.4
C18.4	ICD10CM	Malignant neoplasm of transverse colon	1	1	69	3	4.3
C20	ICD10CM	Malignant neoplasm of rectum	1	1	362	15	4.1
C18.5	ICD10CM	Malignant neoplasm of splenic flexure	1	1	30	1	3.3
153.6	ICD9CM	Malignant neoplasm of ascending colon	1	1	125	4	3.2
153.1	ICD9CM	Malignant neoplasm of transverse colon	1	1	35	1	2.9
D37.3	ICD10CM	Neoplasm of uncertain behavior of appendix	0	1	37	1	2.7
153.5	ICD9CM	Malignant neoplasm of appendix vermiformis	1	1	42	1	2.4
C19	ICD10CM	Malignant neoplasm of rectosigmoid junction	1	1	302	7	2.3
C18.3	ICD10CM	Malignant neoplasm of hepatic flexure	1	1	47	1	2.1
D37.4	ICD10CM	Neoplasm of uncertain behavior of colon	0	1	48	1	2.1
C18.9	ICD10CM	Malignant neoplasm of colon, unspecified	1	1	979	18	1.8
C18.2	ICD10CM	Malignant neoplasm of ascending colon	1	1	238	4	1.7
153.3	ICD9CM	Malignant neoplasm of sigmoid colon	1	1	125	2	1.6
153.9	ICD9CM	Malignant neoplasm of colon, unspecified site	1	1	632	10	1.6
153.8	ICD9CM	Malignant neoplasm of other specified sites of large intestine	1	1	69	1	1.4
154	ICD9CM	Malignant neoplasm of rectosigmoid junction	1	1	167	2	1.2
C18	ICD10CM	Malignant neoplasm of colon	1	1	484	6	1.2
C18.1	ICD10CM	Malignant neoplasm of appendix	1	1	89	1	1.1
C18.7	ICD10CM	Malignant neoplasm of sigmoid colon	1	1	231	1	0.4
153	ICD9CM	Malignant neoplasm of hepatic flexure	1	1	40	0	0
153.2	ICD9CM	Malignant neoplasm of descending colon	1	1	37	0	0
153.7	ICD9CM	Malignant neoplasm of splenic flexure	1	1	13	0	0
154	ICD9CM	Malignant neoplasm of rectum rectosigmoid junction and anus	1	0	6	0	0
159	ICD9CM	Malignant neoplasm of intestinal tract, part unspecified	1	0	38	0	0
209.1	ICD9CM	Malignant carcinoid tumors of the appendix, large intestine, and rectum	1	0	0	0	0
209.11	ICD9CM	Malignant carcinoid tumor of the appendix	1	0	8	0	0
209.12	ICD9CM	Malignant carcinoid tumor of the cecum	1	0	5	0	0
209.13	ICD9CM	Malignant carcinoid tumor of the ascending colon	1	0	0	0	0
209.14	ICD9CM	Malignant carcinoid tumor of the transverse colon	1	0	0	0	0
209.15	ICD9CM	Malignant carcinoid tumor of the descending colon	1	0	1	0	0
209.16	ICD9CM	Malignant carcinoid tumor of the sigmoid colon	1	0	1	0	0
209.17	ICD9CM	Malignant carcinoid tumor of the rectum	1	0	5	0	0
230.3	ICD9CM	Carcinoma in situ of colon	1	1	71	0	0
230.4	ICD9CM	Carcinoma in situ of rectum	1	1	6	0	0
796.7	ICD9CM	Abnormal cytologic smear of anus and anal HPV	1	0	1	0	0
C18.0	ICD10CM	Malignant neoplasm of cecum	1	1	115	0	0
C18.6	ICD10CM	Malignant neoplasm of descending colon	1	1	79	0	0
C21	ICD10CM	Malignant neoplasm of anus and anal canal	1	0	0	0	0
C21.2	ICD10CM	Malignant neoplasm of cloacogenic zone	1	0	2	0	0
C26.0	ICD10CM	Malignant neoplasm of intestinal tract, part unspecified	1	0	16	0	0
C7A.020	ICD10CM	Malignant carcinoid tumor of the appendix	1	0	21	0	0
C7A.021	ICD10CM	Malignant carcinoid tumor of the cecum	1	0	6	0	0
C7A.022	ICD10CM	Malignant carcinoid tumor of the ascending colon	1	0	1	0	0
C7A.023	ICD10CM	Malignant carcinoid tumor of the transverse colon	1	0	1	0	0
C7A.025	ICD10CM	Malignant carcinoid tumor of the sigmoid colon	1	0	2	0	0
C7A.026	ICD10CM	Malignant carcinoid tumor of the rectum	1	0	16	0	0
C7A.029	ICD10CM	Malignant carcinoid tumor of the large intestine, unspecified portion	1	0	8	0	0
D01.0	ICD10CM	Carcinoma in situ of colon	1	1	38	0	0
D01.1	ICD10CM	Carcinoma in situ of rectosigmoid junction	1	1	0	0	0
D01.2	ICD10CM	Carcinoma in situ of rectum	1	1	6	0	0
R85.614	ICD10CM	Cytologic evidence of malignancy on smear of anus	1	0	0	0	0
V10.05	ICD9CM	Personal history of malignant neoplasm of large intestine	1	1	0	0	0
V10.06	ICD9CM	Personal history of malignant neoplasm of rectum, rectosigmoid junction, and anus	1	0	0	0	0
Z85.02	ICD10CM	Personal history of malignant neoplasm of stomach	1	0	0	0	0
Z85.03	ICD10CM	Personal history of malignant neoplasm of large intestine	1	1	0	0	0
Z85.038	ICD10CM	Personal history of other malignant neoplasm of large intestine	1	1	0	0	0
Z85.048	ICD10CM	Personal history of other malignant neoplasm of rectum, rectosigmoid junction, and anus	1	0	0	0	0

We also present the 2x2 tables and crude odds ratios between these two different definitions of colorectal cancer and HIV status in **Table S6**. The crude odds ratio in the time-restricted phenome (t = 1) is 10.29 using version 1.2 colorectal cancer mapping and 0.32 using version X mapping.

Table S6. Colorectal cancer and HIV contingency table by phecode mapping version and crude odds ratio

	**Colorectal cancer**			
	**Version 1.2**		**Version X**	
**HIV**	1	0	1	0
1	478	108	60	526
0	2611	6069	2305	6375
**Crude odds ratio**	10.29		0.32	

Other hits in the infectious disease category like HIV infection, symptomatic (phecode 071.1) and viral warts & HPV (phecode 078) share many of the same underlying ICD codes as with phecode 071, which implies similar overlap with colorectal cancer as defined by phecode mapping table version 1.2.

References

1. Beesley LJ, Mukherjee B. Case studies in bias reduction and inference for electronic health record data with selection bias and phenotype misclassification. *Stat Med*. 2022;41(28):5501-5516. doi:10.1002/sim.9579

2. Robins JM, Rotnitzky A. Semiparametric Efficiency in Multivariate Regression Models with Missing Data. *J Am Stat Assoc*. 1995;90(429):122-129. doi:10.1080/01621459.1995.10476494

3. Zivich PN, Breskin A. Machine Learning for Causal Inference: On the Use of Cross-fit Estimators. *Epidemiology*. 2021;32(3):393-401. doi:10.1097/EDE.0000000000001332

4. Zhong Y, Kennedy EH, Bodnar LM, Naimi AI. AIPW: An R Package for Augmented Inverse Probability–Weighted Estimation of Average Causal Effects. *Am J Epidemiol*. 2021;190(12):2690-2699. doi:10.1093/aje/kwab207

5. Lumley T. Post-stratification, raking, and calibration. In: *Complex Surveys: A Guide to Analysis Using R*. John Wiley & Soncs; 2010:135-156.

6. Kundu R, Shi X, Morrison J, Mukherjee B. A Framework for Understanding Selection Bias in Real-World Healthcare Data. Published online 2023. doi:10.48550/ARXIV.2304.04652

7. Beesley LJ, Mukherjee B. Statistical inference for association studies using electronic health records: handling both selection bias and outcome misclassification. *Biometrics*. Published online December 3, 2020:biom.13400. doi:10.1111/biom.13400

8. Kim S. ppcor: An R Package for a Fast Calculation to Semi-partial Correlation Coefficients. *Commun Stat Appl Methods*. 2015;22(6):665-674. doi:10.5351/CSAM.2015.22.6.665

9. Salvatore M, Beesley LJ, Fritsche LG, et al. Phenotype risk scores (PheRS) for pancreatic cancer using time-stamped electronic health record data: Discovery and validation in two large biobanks. *J Biomed Inform*. Published online December 2020:103652. doi:10.1016/j.jbi.2020.103652

10. Lumley T. CRAN - Package survey. Published 2023. Accessed August 11, 2023. https://cran.r-project.org/web/packages/survey/index.html

11. van Alten S, Domingue BW, Galama T, Marees AT. *Reweighting the UK Biobank to Reflect Its Underlying Sampling Population Substantially Reduces Pervasive Selection Bias Due to Volunteering*. Epidemiology; 2022. doi:10.1101/2022.05.16.22275048

12. White A, Ironmonger L, Steele RJC, Ormiston-Smith N, Crawford C, Seims A. A review of sex-related differences in colorectal cancer incidence, screening uptake,

routes to diagnosis, cancer stage and survival in the UK. *BMC Cancer*. 2018;18(1):906. doi:10.1186/s12885-018-4786-7

13. Van Alten S, Domingue BW, Faul J, Galama TJ, Marees AT. Should Representativeness Be Avoided? Reweighting the UK Biobank Corrects for Pervasive Selection Bias Due to Volunteering. *SSRN Electron J*. Published online 2023. doi:10.2139/ssrn.4493234

14. Beesley LJ, Salvatore M, Fritsche LG, et al. The emerging landscape of health research based on biobanks linked to electronic health records: Existing resources, statistical challenges, and potential opportunities. *Stat Med*. 2020;39(6):773-800. doi:10.1002/sim.8445

15. Weiskopf NG, Weng C. Methods and dimensions of electronic health record data quality assessment: enabling reuse for clinical research. *J Am Med Inform Assoc*. 2013;20(1):144-151. doi:10.1136/amiajnl-2011-000681

16. Farmer R, Mathur R, Bhaskaran K, Eastwood SV, Chaturvedi N, Smeeth L. Promises and pitfalls of electronic health record analysis. *Diabetologia*. 2018;61(6):1241-1248. doi:10.1007/s00125-017-4518-6

17. Bots SH, Groenwold RHH, Dekkers OM. Using electronic health record data for clinical research: a quick guide. *Eur J Endocrinol*. 2022;186(4):E1-E6. doi:10.1530/EJE-21-1088

18. Callahan A, Shah NH, Chen JH. Research and Reporting Considerations for Observational Studies Using Electronic Health Record Data. *Ann Intern Med*. 2020;172(11_Supplement):S79-S84. doi:10.7326/M19-0873

19. Cyganek B, Graña M, Krawczyk B, et al. A Survey of Big Data Issues in Electronic Health Record Analysis. *Appl Artif Intell*. 2016;30(6):497-520. doi:10.1080/08839514.2016.1193714

20. Streeter AJ, Lin NX, Crathorne L, et al. Adjusting for unmeasured confounding in nonrandomized longitudinal studies: a methodological review. *J Clin Epidemiol*. 2017;87:23-34. doi:10.1016/j.jclinepi.2017.04.022

21. Zhang L, Wang Y, Schuemie MJ, Blei DM, Hripcsak G. Adjusting for indirectly measured confounding using large-scale propensity score. *J Biomed Inform*. 2022;134:104204. doi:10.1016/j.jbi.2022.104204

22. Hubbard RA, Tong J, Duan R, Chen Y. Reducing Bias Due to Outcome Misclassification for Epidemiologic Studies Using EHR-derived Probabilistic Phenotypes. *Epidemiology*. 2020;31(4):542-550. doi:10.1097/EDE.0000000000001193

23. Tong J, Huang J, Chubak J, et al. An augmented estimation procedure for EHR-based association studies accounting for differential misclassification. *J Am Med Inform Assoc JAMIA*. 2020;27(2):244-253. doi:10.1093/jamia/ocz180

24. Getzen E, Ungar L, Mowery D, Jiang X, Long Q. Mining for equitable health: Assessing the impact of missing data in electronic health records. *J Biomed Inform*. 2023;139:104269. doi:10.1016/j.jbi.2022.104269

25. Haneuse S, Arterburn D, Daniels MJ. Assessing Missing Data Assumptions in EHR-Based Studies: A Complex and Underappreciated Task. *JAMA Netw Open*. 2021;4(2):e210184. doi:10.1001/jamanetworkopen.2021.0184

26. Haneuse S, Bogart A, Jazic I, et al. Learning About Missing Data Mechanisms in Electronic Health Records-based Research: A Survey-based Approach. *Epidemiology*. 2016;27(1):82-90. doi:10.1097/EDE.0000000000000393

27. Weiskopf NG, Rusanov A, Weng C. Sick patients have more data: the non-random completeness of electronic health records. *AMIA Annu Symp Proc AMIA Symp*. 2013;2013:1472-1477.

28. Seaman SR, White IR. Review of inverse probability weighting for dealing with missing data. *Stat Methods Med Res*. 2013;22(3):278-295. doi:10.1177/0962280210395740

29. Pedersen A, Mikkelsen E, Cronin-Fenton D, et al. Missing data and multiple imputation in clinical epidemiological research. *Clin Epidemiol*. 2017;Volume 9:157-166. doi:10.2147/CLEP.S129785

30. Li L, Shen C, Li X, Robins JM. On weighting approaches for missing data. *Stat Methods Med Res*. 2013;22(1):14-30. doi:10.1177/0962280211403597

31. Haneuse S, Daniels M. A General Framework for Considering Selection Bias in EHR-Based Studies: What Data are Observed and Why? *EGEMs Gener Evid Methods Improve Patient Outcomes*. 2016;4(1):16. doi:10.13063/2327-9214.1203

32. Peskoe SB, Arterburn D, Coleman KJ, Herrinton LJ, Daniels MJ, Haneuse S. Adjusting for selection bias due to missing data in electronic health records-based research. *Stat Methods Med Res*. 2021;30(10):2221-2238. doi:10.1177/09622802211027601

33. Weiskopf NG, Dorr DA, Jackson C, Lehmann HP, Thompson CA. Healthcare utilization is a collider: an introduction to collider bias in EHR data reuse. *J Am Med Inform Assoc*. Published online February 8, 2023:ocad013. doi:10.1093/jamia/ocad013

34. Rusanov A, Weiskopf NG, Wang S, Weng C. Hidden in plain sight: bias towards sick patients when sampling patients with sufficient electronic health record data for research. *BMC Med Inform Decis Mak*. 2014;14(1):51. doi:10.1186/1472-6947-14-51

35. Wei WQ, Bastarache LA, Carroll RJ, et al. Evaluating phecodes, clinical classification software, and ICD-9-CM codes for phenome-wide association studies in the electronic health record. Rzhetsky A, ed. *PLOS ONE*. 2017;12(7):e0175508. doi:10.1371/journal.pone.0175508

36. Liu X, Chubak J, Hubbard RA, Chen Y. SAT: a Surrogate-Assisted Two-wave case boosting sampling method, with application to EHR-based association studies. *J Am Med Inform Assoc*. 2022;29(5):918-927. doi:10.1093/jamia/ocab267

37. Yin Z, Tong J, Chen Y, Hubbard RA, Tang CY. A cost-effective chart review sampling design to account for phenotyping error in electronic health records (EHR) data. *J Am Med Inform Assoc*. 2021;29(1):52-61. doi:10.1093/jamia/ocab222

38. Teixeira PL, Wei WQ, Cronin RM, et al. Evaluating electronic health record data sources and algorithmic approaches to identify hypertensive individuals. *J Am Med Inform Assoc*. 2017;24(1):162-171. doi:10.1093/jamia/ocw071

39. Li R, Chen Y, Moore JH. Integration of genetic and clinical information to improve imputation of data missing from electronic health records. *J Am Med Inform Assoc*. 2019;26(10):1056-1063. doi:10.1093/jamia/ocz041

40. Ma Y, Patil S, Zhou X, Mukherjee B, Fritsche LG. ExPRSweb: An online repository with polygenic risk scores for common health-related exposures. *Am J Hum Genet*. 2022;109(10):1742-1760. doi:10.1016/j.ajhg.2022.09.001

41. Westreich D, Edwards JK, Lesko CR, Cole SR, Stuart EA. Target Validity and the Hierarchy of Study Designs. *Am J Epidemiol*. 2019;188(2):438-443. doi:10.1093/aje/kwy228

42. Goldstein BA, Bhavsar NA, Phelan M, Pencina MJ. Controlling for Informed Presence Bias Due to the Number of Health Encounters in an Electronic Health Record. *Am J Epidemiol*. 2016;184(11):847-855. doi:10.1093/aje/kww112

43. Agniel D, Kohane IS, Weber GM. Biases in electronic health record data due to processes within the healthcare system: retrospective observational study. *BMJ*. Published online April 30, 2018:k1479. doi:10.1136/bmj.k1479

44. Phelan M, Bhavsar N, Goldstein BA. Illustrating Informed Presence Bias in Electronic Health Records Data: How Patient Interactions with a Health System Can Impact Inference. *EGEMs Gener Evid Methods Improve Patient Outcomes*. 2017;5(1):22. doi:10.5334/egems.243

45. Harton J, Mitra N, Hubbard RA. Informative presence bias in analyses of electronic health records-derived data: a cautionary note. *J Am Med Inform Assoc*. Published online April 19, 2022:ocac050. doi:10.1093/jamia/ocac050

46. Sisk R, Lin L, Sperrin M, et al. Informative presence and observation in routine health data: A review of methodology for clinical risk prediction. *J Am Med Inform Assoc*. 2021;28(1):155-166. doi:10.1093/jamia/ocaa242

47. O'Neill TJ, Nguemo JD, Tynan AM, Burchell AN, Antoniou T. Risk of Colorectal Cancer and Associated Mortality in HIV: A Systematic Review and Meta-Analysis. *JAIDS J Acquir Immune Defic Syndr*. 2017;75(4):439-447. doi:10.1097/QAI.0000000000001433

48. Coghill AE, Engels EA, Schymura MJ, Mahale P, Shiels MS. Risk of Breast, Prostate, and Colorectal Cancer Diagnoses Among HIV-Infected Individuals in the United States. *JNCI J Natl Cancer Inst*. 2018;110(9):959-966. doi:10.1093/jnci/djy010

49. Carroll RJ, Bastarache L, Denny JC. R PheWAS: data analysis and plotting tools for phenome-wide association studies in the R environment. *Bioinforma Oxf Engl*. 2014;30(16):2375-2376. doi:10.1093/bioinformatics/btu197

50. PheWAS. Published online May 5, 2023. Accessed May 5, 2023. https://github.com/PheWAS/PheWAS

51. Shuey M, Stead W, Aka I, et al. *Next-Generation Phenotyping: Introducing PhecodeX for Enhanced Discovery Research in Medical Phenomics*. Health Informatics; 2023. doi:10.1101/2023.06.18.23291088

52. PheWAS/PhecodeX. Accessed August 11, 2023. https://github.com/PheWAS/PhecodeX

53. Dandapani SV, Eaton M, Thomas CR, Pagnini PG. HIV- positive anal cancer: an update for the clinician. *J Gastrointest Oncol*. 2010;1(1):34-44. doi:10.3978/j.issn.2078-6891.2010.005
